# Reconstructing recent human phylogenies with forensic STR loci: A statistical approach

**DOI:** 10.1186/1471-2156-6-47

**Published:** 2005-09-28

**Authors:** Suraksha Agrawal, Faisal Khan

**Affiliations:** 1 Department of Medical Genetics, Sanjay Gandhi Post Graduate Institute of Medical Sciences, Raebareli Road, Lucknow (UP) 226014 India; 2 Department of Biotechnology, Bundelkhand University, Jhansi (UP), India

**Keywords:** Short tandem repeats, Forensic, Phylogeny, Neighbor-joining, Maximum likelihood, PC plot

## Abstract

**Background:**

Forensic Short Tandem Repeat (STR) loci are effective for the purpose of individual identification, and other forensic applications. Most of these markers have high allelic variability and mutation rate because of which they have limited use in the phylogenetic reconstruction. In the present study, we have carried out a meta-analysis to explore the possibility of using only five STR loci (TPOX, FES, vWA, F13A and Tho1) to carry out phylogenetic assessment based on the allele frequency profile of 20 world population and north Indian Hindus analyzed in the present study.

**Results:**

Phylogenetic analysis based on two different approaches – genetic distance and maximum likelihood along with statistical bootstrapping procedure involving 1000 replicates was carried out. The ensuing tree topologies and PC plots were further compared with those obtained in earlier phylogenetic investigations. The compiled database of 21 populations got segregated and finely resolved into three basal clusters with very high bootstrap values corresponding to three geo-ethnic groups of African, Orientals, and Caucasians.

**Conclusion:**

Based on this study we conclude that if appropriate and logistic statistical approaches are followed then even lesser number of forensic STR loci are powerful enough to reconstruct the recent human phylogenies despite of their relatively high mutation rates.

## Background

Short Tandem Repeats (STR), with a repetitive sequence ranging from 2–6 base pairs are amongst the most polymorphic markers reported till date. They exhibit substantial allelic variability due to high rate of germline mutations [1]. The STR loci have a uniform and dense distribution throughout the genome and exhibit high level of relatively stable polymorphism [2]. All these features makes them an ideal candidate for diverse applications including forensic applications [3], individual identification, paternity/maternity detection [2], fine scale genetic mapping [4] and inter and intra group phylogenetic reconstruction [5].

However, a specific set of STR can be employed for specific applications and this specificity is solely based on the properties of STR loci involved and their suitability to the particular application. STR loci used for forensic purposes are the one that possess numerous observed alleles, high level of heterozygosity, high polymorphism information content and high power of exclusion. On the contrary, STR loci preferred for the phylogenetic analysis of the human populations are those which have substantial lower allelic counts and carries signature alleles for specific populations [6,7]. Still, there are few studies in which there is some overlap between the sets of forensic STRs and those exclusively studied for phylogenetic investigation. However, this overlap is not extensive and is without any definitive rationale or design.

There are two school of thoughts regarding the use of forensic STR in phylogenetic studies. According to one view, the requirement of extremely high level of intra group variation along with high mutation rates in forensic systems indicates a rapid diffusion of genetic variation and thus, confers a greater risk of failure in detection of convergent evolution among some populations [8]. Other perception is that random noise generated by allelic variability in forensic systems is not strong enough to veil the evolutionary signals generated by these STR loci. Furthermore, fine scale resolution of forensic STR may prove handful in delineating genetic difference and affinities between closely related ethnic groups [6].

In the present study, we have made an attempt to explore the utility of forensic STR loci in inferring phylogenetic relationships. To approach this goal, we have compiled a geographically targeted and racially diverse set of 21-population database from forensic literature obtained from Wolfgang Huckenbeck and Hans-Georg Scheil's website "The Distribution of the Human DNA-PCR Polymorphisms" [9] while the north Indian Hindus from the state of Uttar Pradesh were genotyped in our own lab. Forensic STR loci for which the allele frequency data was compiled were Tho1, vWA, FES. F13 and TPOX. They all carries tetrameric core repeat sequence and reside on different chromosomes and are amongst the most reputed one in forensic system. The choice of these loci is exclusively on the basis that these markers have been studied in all the 21 populations hence a precise phylogenetic analysis could be performed [Table 1].

**Table 1:** Population compiled for database of five forensic STR loci

**S.No.**	**Populations**	No. of samples analyzed *				
		
		**TPOX**	**FES**	**vWA**	**F13A**	**Tho1**
1	Basque	768	627	615	208	859
2	Poland	703	643	572	334	488
3	Germany	2876	7683	13667	3438	7373
4	Italy	11388	4827	7135	1677	4900
5	Portugal	1239	2091	4720	1158	4639
6	Spain	1782	2325	3361	1864	4037
7	Canadian Caucasians	435	321	428	435	435
8	Middle Eastern Arabs	165	132	149	127	173
9	US Caucasian	562	597	759	587	765
10	Slovenia	235	235	779	236	560
11	Austria	153	153	1946	1056	1816
12	China	658	435	1146	137	2503
13	Japan	1491	397	1743	668	2905
14	Philippine	498	103	376	133	528
15	Taiwan	716	100	600	149	764
16	Sharawasi Africans	59	59	99	59	59
17	Cameroon	65	65	65	65	65
18	Moroccan Arabs	127	199	193	75	271
19	Lisongo Africans	32	60	30	30	30
20	US Afro-Americans	580	679	797	691	793
21	North Indian Hindus **	1000	1000	1000	1000	1000

Source of data:*  [9]

** Present study

Phylogenetic assessment was carried out through two different approaches – genetic distance and maximum likelihood along with a statistical Bootstrapping procedure involving 1000 replicates. The ensuing tree topologies and PC plots were then compared with those obtained in earlier phylogenetic investigations. The main question that we have tried to address in this meta-analysis is whether a limited number of forensic STR can predict accurate human phylogenies, if the data is evaluated using proper statistical approaches.

## Results

### Allele frequency distribution

Analysis of five STR loci- Tho1, vWA, FES, F13 and TPOX has revealed high level of diversity among North Indian Hindus. Total 7–8 alleles were found (7 each for Tho1, vWA, F13 and TPOX and 8 for FES). All the loci were in Hardy-Weinberg equilibrium. Table 2 shows allele frequency distribution at all the five loci among North Indian Hindus. High allelic variability was further depicted by high-observed Heterozygosity (0.68 at Tho1-0.76 at vWA), high PIC (0.66 at F13-0.74 at Tho1) and high power of exclusion (0.28 at F13-0.38 at Tho1). All these criterions are indicative of the fact that these STR loci are useful and informative tools for all types of forensic applications.

**Table 2:** Different statistical analysis done on allele frequency data of five STR loci which are important criterion for a good forensic loci

	**Tho1**	**VWA**	**FES**	**F13**	**TPOX**
**Allele3**				0.06	
**Allele 4**				0.06	
**Allele 5**	0.01			0.20	
**Allele 6**	0.28			0.40	
**Allele 7**	0.16		0.04	0.26	0.02
**Allele 8**	0.08		0.01	0.02	0.40
**Allele 9**	0.17		0.08		0.12
**Allele 9.3**	0.30				
**Allele 10**	0.06		0.27		0.08
**Allele 11**			0.36		0.32
**Allele 12**			0.18		0.04
**Allele 13**			0.02		0.02
**Allele 14**		0.14	0.04		
**Allele 15**		0.04			
**Allele 16**		0.34			
**Allele 17**		0.14			
**Allele 18**		0.30			
**Allele 19**		0.02			
**Allele 20**		0.02			
**Observed Heterozygosity**	0.680	0.760	0.760	0.740	0.730
**PIC**	0.738	0.714	0.725	0.660	0.669
**Power of exclusion**	0.381	0.354	0.371	0.283	0.308
**Average heterozygosity**	**0.734**				
**Mean PIC**	**0.700**				
**Total exclusionary power**	**0.875**				

### Phylogenetic assessment

**Figure 1:**
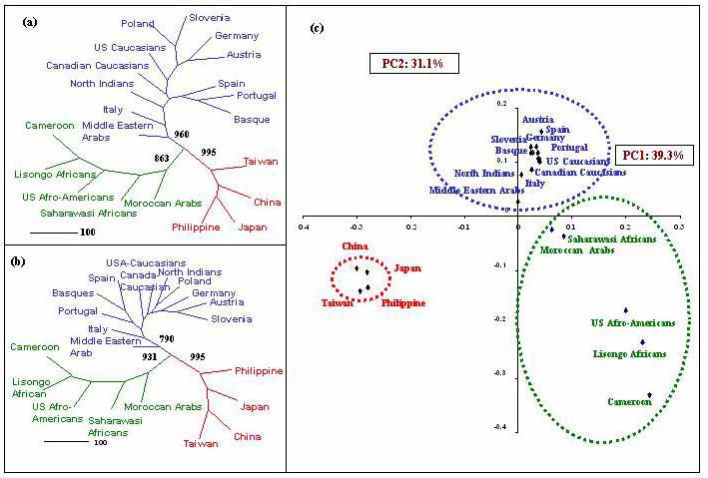
Phylogenetic reconstruction based on (a) Neighbor-joining (NJ) tree with 1000 bootstrap replicates; (b) Maximum Likelihood (ML) tree with 1000 bootstrap replicates and PC plot analysis based on allele frequency differences.

Phylogenetic analysis carried out in 21 populations is depicted by two enrooted radial phylograms (NJ and ML) as shown in Figure 1a and 1b and a PC plot was plotted based on the allele frequency variation [Figure 1c]. The edge lengths displayed in these phylograms indicated that the amount of evolutionary change occurred along each branch. The scores next to the nodes characterize the number of bootstrap replicates (out of 1000) exhibiting these specific bifurcations.

NJ phylogenetic tree depicts three different basal groups corresponding to three ethnic groups- African, Caucasian and Orientals. The Caucasian cluster has three monophyletic units- Austria/German, Basque/Portuguese and USA/Canadian Caucasians. Total 8 out of the 14 nodes have bootstrap values more than 50%, most important among them is (i) division between Africans and other two groups of Caucasians and Orientals-863, (ii) the division between Caucasian and other two groups-960 and (iii) the division between Orientals and other two groups-995.

ML phylograms also displayed comparable topology to that of NJ tree. It has also depicted three basal nodes with clear demarcation of Africans, Caucasians and Orientals. Similar to NJ tree, all the three basal monophyletic topologies in ML tree have bootstrap values more than 50% i.e. 931, 790 and 995 respectively for Africans, Caucasians and Orientals. In both the phylogenies, NJ and ML, North Indian Hindus clustered with the Caucasians albeit with low bootstrap value of 456.

First and second Principal component that together constitutes 70.4% of the total variability (PC1-39.3% and PC2-31.1%) were plotted and presented in Figure 1c. There is a significant separation of the Orientals from Caucasians on Y-axis (PC2) and with that of Africans on X-axis (PC1). While Caucasians and Africans reveal relatively clear division along both X-axis (PC1) and Y-axis (PC2), with Middle Eastern Arabs and Moroccan Arabs positioning near to each other. Furthermore, the African populations have clustered into two sub-groups corresponding to the Central and North Africans. North Indian Hindus have clustered with Caucasians.

## Discussion

Five forensic STR loci are found highly successful in providing fine resolution for the reconstruction of recent human evolutionary histories. All three approaches used for phylogenetic reconstruction (NJ and ML tree topologies and PC-plot analysis) have depicted strong racial partitioning and deciphering the accurate phylogenetic information about North Indian Hindus which is in accordance with those derived from other more renounced phylogenetic markers as well as historical evidences [10-12].

The phylograms (NJ and ML) generated from present data set were calculated from CONTML and NJ algorithms, where CONTML works upon the conjecture that random action of genetic drift is the solitary basis of the differences between allele frequencies in different population groups [13]. On the contrary, the NJ algorithm construct a branching array from a matrix of genetic distances calculated from Nei's formula assuming that both genetic drift and mutation causes allele frequency differences [14].

Both phylograms, NJ and ML have more or less similar basal cluster patterns among the three geo-ethnic groups indicating that component of genetic drift instead of mutation is the major player in these distant estimates. Both the trees have longer African branch than any other group. Such a patristic separation is also visible in PC-plot analysis [Figure 1c]. The African populations have been clustered into central (Cameroon and Lisongo) and North African (Moroccan Arabs and Saharawasi Africans) groups. Such clustering has also been reported by Cavalli-Sforza et al, 2003 based Fst genetic distance based on polymorphisms of 120 protein-coding genes [15] and Y-chromosome binary haplogroup [16,17]. This sub clustering further strengthens the utility of the 5 STR loci in deciphering the accurate phylogenies even within the same geographical region. Middle Eastern Arabs display a branch nearer to Caucasians and to some extent near to Moroccan Arabs suggesting strong Caucasian element along with African admixtures suggestive of the Demic expansion of the middle east genes, agriculture innovations and languages into north west Africa [16,17], which is further supported by the near medial position of Arabs in the PC-plot. Recently, Y-chromosome SNP analysis by Al-Zahery et al. 2003 [18] has also revealed similar pattern in other Middle Eastern populations. European branching pattern differs slightly between the two trees, but still both are resolving completely with Basque, Spaniards and Portuguese having a separate cluster from that of German/Austrian branch. North Indian Hindus clustered with Caucasians in both the phylograms, which is in agreement with the findings of earlier studies. Bamshad et al. 2001[12] based on mtDNA HVR-I and HVR-II sequencing and Y-chromosome haplotypes has shown that North Indian populations reveal high frequency of west Eurasian haplogroups. North Indian Hindus are basically Indo-Aryan speakers, who invaded from the steppes of central Asia and then settled in the Indus valley, in northwestern India [10].

The major finding of the present study is the productivity of a limited set of 5 forensic STR loci in resolving the human phylogenies in a similar manner as reported elsewhere on a much higher number of loci. Further, the study also highlights the utility of combined use of varied statistical approaches in reaching a definitive conclusion. Our study scores a point over some of the successful reports like that of Bowcock et al., 1997[19] which has shown substantial phylogenies but with much larger sets of STR – 30 STR loci. Similarly, Perez-lezaun et al. 1999 [20] has used 20 STR loci and computed Fst based distances depicting similar separation of inter and intra ethnic groups. Even though, in the same study, phylogenetic tree based on D_SW _distance exhibited a defused picture having trifurcation two Caucasoid and one African group.

Various attempts of phylogenetic reconstruction using forensic STR loci have also been done in recent past like that of Budowle and Chakraborty, 2001[21] who studied 13 CODIS loci, but their phylogenetic assessment was confined to simple NJ and UPGMA trees and distance measures which yields single output tree. To overcome this, we have incorporated both the phylogenetic approaches i.e. distance and optimal criterion along with statistical bootstrapping which yields 1000 trees and then built a consensus tree. In this regard, a successful attempt was made by Rowold et al. 2003 [6], by compiling 10 geographically and racially different populations on five forensic STR loci. However, incorporating different set of STR loci, we have been able to compile larger population database of 21 populations.

Overall, the analysis of five forensic STR loci have depicted a strong racial phylogeny indicating that high heterozygosity and/or numerous observed alleles do not necessarily interfere with the phylogenetic information content of the locus, provided that frequency distribution of the populations is significantly different. Significantly, larger number of alleles increases the chances of the presence of signature alleles in segregating populations. Despite all the potential problems associated with forensic STR loci including that of high mutation rates, successfully resolution the genetic difference between inter and intra geo-ethnic groups suggesting that if well-defined statistical approaches are followed, then even a smaller number of forensic STR loci are powerful enough in reconstructing human phylogenies.

## Methods

### Populations (North Indian Hindus)

A total of 1000 unrelated individuals were randomly selected. Regional addresses and detailed computerized lists were prepared before sample collection. Random numbers were generated with the help of computer and samples were collected from the different collection sites of Uttar Pradesh- Lucknow, Kanpur, Faizabad, Basti, Gonda and Agra. Whole blood was obtained by venipuncture and collected in EDTA vacutainer tubes. Three-generation pedigree charts were prepared to assure un-relatedness in all the samples. The ethical committee of the institute approved the study and blood samples were taken after obtaining informed consent from the subjects.

### DNA extraction and STR genotyping

DNA was extracted by phenol chloroform method as described by Comey et al. 1993 [22] and purified by ethanol precipitation. All the five STR loci were detected by PCR. PCR amplification was performed using flanking primers described elsewhere [20]. The amplified product was separated and detected on 9% PAGE using silver staining.

### Population database

20 geographically targeted populations were selected from forensic literature [9], while the data of north Indian Hindus was generated from our lab (Agrawal et al., unpublished data). The criterion of selection was to cover the major geographical and geo-ethnic groups i.e. African, Caucasoid and Orientals. All the populations selected have allele frequency data for five STR loci. In order to embrace a large sample size and to overcome the predicament of some studies focusing only on 2 or 3 STR loci, allele frequency profile of different STR loci analyzed in different populations samples but of the same geographic or ethnic origin has also been included. However, wherever possible, a care has been taken to include the allele frequency profile of the same set of sample for different markers. For example, same 65 samples of Cameroon population has been used for allele frequency data of 5 STR, whereas a large pooled sample size has been used for other groups like Germany, Portugal, Italy, China, Japan etc. In order to avoid the discrepancies, number of samples for each population genotyped for different STR loci and source of allele frequency data is shown in Table 1. Maximum of the populations compiled in the database are pooled samples from different parts of that country. Notably, Sardinians are excluded from Italians, Azores from Portugal and Canary Island from Spain. Middle Eastern Arabs included Arabs mainly from Saudia Arabia, Qatar and Yemen.

### Statistical analysis

Allele frequencies were calculated by a simple gene count method. A total analysis was executed based upon the allelic frequency distribution of the five STR. Heterozygosity, HWE, PIC and power of exclusion was calculated using Cervus v1 [23]. Further, Statistical analysis was executed based upon the allelic frequency distribution of the five STR. A 1000 replicate bootstrap data was generated from SEQBOOT option in PHYLIP version 3.5c [13]. Distance values were estimated using Nei's formula [14], and a phylogeny was inferred by the neighbor joining (NJ) option in PHYLIP version 3.5c [13]. Phylogenetic reconstruction was also done based on maximum likelihood (ML) and the STR frequency distribution (CONTML in PHYLIP version 3.5c)[13]. Finally, a principal component (PC) analysis was generated by POPSTR and first and second PC was plotted as described elsewhere [24].

## Authors' contributions

SA has conceptualized the paper provided important intellectual inputs in intrepretation of data and preparation of the manuscript and FK carried out statistical analysis and drafted the manuscript. Both authors read and approved the final manuscript
